# Questions and Answers

**Published:** 1895-07

**Authors:** 


### Editorial.

### QUESTIONS AND ANSWERS.

There has arisen in the past few years in dentistry a method, peculiar to itself and it is believed to this country, of interrogating individuals upon professional problems for the benefit of dental societies, journals, and individuals. These generally are limited to some special practice, but frequently are extended into the domain of pathology, bacteriology, and histology.

This method of arriving at truth has its good and bad side, the latter promising to overcome the former and become a serious menace to more thorough work.

It had its origin in the, perhaps, laudable idea of concentrating the practice of many men and deducing therefrom a positive method, and thereby settling many pathological and therapeutical difficulties. To accomplish this parties have been delegated by certain journals to interview teachers in colleges and others by letter in which questions were enclosed. The recipients were supposed to have sufficient love of approbation and desire to aid their fellow-practitioners to induce them to sacrifice a portion of their valuable time to enlighten the world as to their methods of practice in peculiar cases. This altruistic work being duly appreciated, the idea extended to other individuals and journals, until the whole matter has become a serious burden in every direction.

Having passed through the individual and local society stage, this system finally reached the dignity of national consideration, and, very naturally, was adopted by the American Dental Association as part of its work. It was presumed that through this a consensus of opinion could be obtained which would result in the solution of many problems in practice. A committee was appointed from this body to prepare questions to be sent down to subordinate societies, and to be discussed at the regular meetings during the winter. It was expected that a condensed summary of these discussions would be made, and the result furnished the members of the American Dental Association at a subsequent meeting.

The result of this action has been a curious admixture of fact and verbiage, with absolutely no conclusions worthy the name. We have read many of these discussions with a commingled feeling of amusement and regret, and it is difficult to decide which one of these is the most pronounced. It is certainly amusing to note the anxiety manifested to present "our peculiar method of practice," forgetting apparently that, after all, it is not alone through this antiseptic, astringent, or escharotic that conditions are to be met, but rather by an intelligent conception of the philosophy which must govern all treatment. The regret must be felt that so much valuable strength is put forth with such unsatisfactory results.

While these questions have been annually sent out by the American Dental Association, without much good having been accomplished, there has been a result of positive value, that of binding subordinate bodies to the parent organization, but whether this is sufficient to warrant a continuance must be left to the good judgment of the parties interested.

If the dental world anticipated any great results from this method of arriving at truth, there must have been by this time a positive disappointment experienced. No one has been enlightened, we imagine, as to the best course to pursue in pulp treatment or the proper method to pursue in sterilization by the discussion, nor are we any nearer the truth than we were at the beginning. We read with interest the plans adopted by our colleagues, and generally end by continuing our own.

It is possible to have a series of questions result in positive value, but the method of answering should be changed. We can conceive of a system by which questions propounded by the American Dental Association might be answered by certain committees,—the committees given power to expend necessary funds to carry on investigations throughout the year. If this work were faithfully performed, there can be no doubt but that the end of the year would find a decidedly more intelligent conception of various subjects. The American Dental Association has expended a considerable sum in valuable work under the direction of the late lamented Dr. Patrick, and this might be extended to other investigations. We can imagine a committee that would do the work thoroughly, and while, possibly, not reaching definite conclusions on all points would, at least, give us an intelligent basis for future work.

The time is approaching for the annual gatherings, and it would be well to have the main question discussed, whether or not the present method has not had its day? The answer to this, it seems to us, should be in the affirmative. As a transition means it has served a good purpose, but to arrive at truth we must question nature in her deeper mysteries, and the answer we will receive will be more satisfactory than that formulated on the "I think" or "I believe" principle, so generally adopted in treating intricate problems.

If societies, journals, and individuals would cease wasting time in this vain pursuit after knowledge and adopt the only true way, positive investigation, intelligence would be increased, and the dental profession would far more rapidly advance to the domain of positive knowledge.

